# Malaria Knowledge and Associated Factors Towards Mosquito Net Use among School Children in Muheza District, Tanzania

**DOI:** 10.24248/eahrj.v9i2.855

**Published:** 2025-12-24

**Authors:** Raphael Hillary Sebukoto, Loveness Urio, Billy Ngasala

**Affiliations:** a National Institute for Medical Research (NIMR), Tanga Centre, Tanzania; b Tanzania Field Epidemiology and Laboratory Training Program (TFELTP); c Muhimbili University of Health and Allied Sciences (MUHAS), Dar-es-Salaam, Tanzania

## Abstract

**Background:**

Schoolchildren face a high risk of malaria, yet remain a neglected demographic in control programmes compared to pregnant women and children under five. In Tanzania, in recent years, mosquito nets have been provided to primary schoolchildren, but usage remains low. Studies on malaria knowledge and mosquito net use among schoolchildren is limited. This study aimed to assess malaria knowledge and identify factors associated with mosquito net use among schoolchildren in Muheza District, Tanzania.

**Methods:**

A school-based cross-sectional study was conducted from December 2022 to February 2023. Schoolchildren and household heads were interviewed using an interviewer-administered questionnaire. Malaria knowledge was assessed, with scores above 50% classified as good knowledge. Modified Poisson regression analysis was used to identify factors associated with mosquito net use, with statistical significance set at *p* ≤ .05.

**Results:**

A total of 530 schoolchildren were enrolled. Of these, 87.2% owned at least one mosquito net, and among them, 69.8% reported using a net the previous night. Overall, 90.6% of children demonstrated good malaria knowledge. Factors significantly associated with mosquito net use included urban residence (aPR 1.40; 95% CI, 1.19 to 1.62), living with parents (aPR 1.35; 95% CI, 1.14–1.58), and ownership of a television or radio (aPR 1.39; 95% CI, 1.05 to 1.83). Household-level determinants included access to electricity (aPR 2.17; 95% CI, 1.12 to 4.20), a household size-to-bed-net ratio of ≤ 2 (aPR 2.30; 95% CI, 1.36 to 3.88), and possession of a mosquito net that had been in use for ≤ 3 years (aPR 1.69; 95% CI, 1.19 to 2.41).

**Conclusion:**

Although most schoolchildren in Muheza District have good malaria knowledge, 30% did not use a mosquito net the night before the interview, and 3% still believed in local herbal remedies. Higher mosquito net use was associated with living in urban areas, parental care, ownership of mass-media devices, household access to electricity, a favourable household size-to-bed-net ratio, and having a relatively new mosquito net. Strengthened malaria education and targeted interventions, particularly in rural areas are urgently needed to promote consistent net use and correct misconceptions regarding malaria treatment.

## BACKGROUND

Malaria remains a major global public health challenge, with an estimated 95% of cases occurring in African countries in 2020. Despite global efforts towards malaria control, malaria-related deaths increased by 12% compared to the previous year^1^, underscoring setbacks in prevention programmes. Children under five years remain the most vulnerable group, accounting for nearly 67% of malaria-related deaths worldwide.^2^ Recent literature shows that schoolchildren are more affected than children under five.^3^ Although schoolchildren constitute over 60% of the reservoir for malaria transmission, they have received less emphasis in malaria prevention efforts.^4^

Regionally, the African continent continues to bear the heaviest burden of malaria globally, accounting for over 90% of malaria cases and deaths worldwide.^1^ Despite considerable progress in the use of different interventions such as insecticide-treated nets (ITNs), indoor residual spraying (IRS), and effective case management, the continent continues to face multiple challenges, such as insecticide resistance, limited health infrastructure, and varying intervention coverage.^5, 6^ Within Africa, the East African Community (EAC) member states; Burundi, Kenya, Rwanda, South Sudan, Tanzania, and Uganda, experience heterogeneous malaria transmission patterns due to ecological diversity and socio-economic factors.^7^ Although several countries in the EAC have incorporated integrated malaria control strategies that align with the World Health Organization (WHO) recommendations, transmission remains high in many areas, particularly in border regions and high-transmission zones where outdoor and asymptomatic infections sustain parasite reservoirs.^8^

At the country level, Tanzania continues to struggle with malaria transmission, especially in high-endemic zones such as Muheza district. Recent studies report that approximately 70% of school-aged children in the district act as reservoirs for ongoing community transmission.^9^ Schoolchildren, who have received less attention in malaria prevention programmes that focus largely on children under five and pregnant women, contribute significantly to the overall malaria burden. This higher prevalence has been linked to control strategies that primarily target adults, who then prioritize the use of mosquito nets for children under five.^10^

Studies across Africa demonstrate various predictors of mosquito net utilisation among schoolchildren. For example, in Malawi, a study reported that a lower household-to-bednet ratio and a higher proportion of nets per household significantly contributed to mosquito net use among schoolchildren.^11^ Similarly, parental educational status and household factors were found to be associated with mosquito net use among schoolchildren in Kenya.^12^ Likewise, in Ethiopia, urban residence, parental literacy, and parental employment were associated with an increased likelihood of mosquito net use among schoolchildren.^4^

In Muheza district, despite high ownership of mosquito nets, usage among schoolchildren remains low^13^, highlighting an important gap in malaria prevention efforts. Notably, 26.4% of schoolchildren in Muheza attend school with asymptomatic malaria infections, contributing to their absenteeism.^13^ However, most malaria control programmes target adults, despite the important role schoolchildren play in play in sustaining community transmission.^14^ Given these findings, assessing baseline knowledge and factors associated with mosquito net use among schoolchildren in Muheza district is essential to inform targeted interventions aimed at reducing malaria transmission.

## METHODS

### Study Design, Site, and Population

This school-based cross-sectional study was conducted in Muheza District, located in the northeastern part of Tanzania, from December 2022 to January 2023. The study population consisted of assented children in Classes IV to VII, selected from four primary schools: Masuguru, Kwambutu, Matombo, and Heinkele. Collectively, these schools had a total of 2,119 children in the target classes. Children who either declined to assent or whose parents declined consent were excluded.

### Sample Size

The sample size was calculated using a standard formula for prevalence studies ^15^, with a 95% confidence level and a 5% Margin of error. Considering the prevalence of 26.4%^13^, Z-score of 1.96, accounting for a 10% non-response rate, and a design effect of 1.6 ^16^, the estimated sample size was 530.

### Sampling Procedures

A list of 113 primary schools was obtained from the Muheza District Council officials. The schools were stratified based on their locality as urban or rural. Two schools were randomly selected from each stratum. Probability proportional to size (PPS) was used to determine the number of children to be sampled from each school. Similarly, the PPS was used to allocate the number of children to be sampled within each class at the selected schools. A systematic sampling method was then used to select schoolchildren from each class, out of whom 20% were visited at home to interview their parents.

### Data Collection

An interviewer-administered questionnaire was used to collect information on demographic characteristics, malaria knowledge and mosquito net use from the study population. Two separate questionnaires were administered: one specifically for schoolchildren and another for their parents or caregivers.. Interviews with schoolchildren were conducted at school during break times to avoid disrupting lessons, while parents and caregivers were interviewed at their homes. The questionnaires were initially developed in English and then translated into Kiswahili, with back-translation into English by an independent person to ensure consistency of meaning. Before the main study, the questionnaires were pre-tested at Ngomeni primary school not included in the sample. Each interview lasted approximately 25 to 30 minutes. Research assistants received training specific to the study protocol and were responsible for data collection.

### Assessment of Malaria Knowledge

Malaria knowledge was evaluated using four indicators: understanding the mode of transmission, recognition of symptoms, familiarity with treatment options, and awareness of preventive measures. Knowledge score was calculated by summing points across the four areas. Participants received a score of one if they correctly identified a mosquito bite as the mode of transmission; otherwise, they scored zero. For symptoms, one point was awarded for each correctly identified key symptom, which included fever, headache, chills, abdominal pain, and vomiting. Regarding treatment, one point was given for each correctly named malaria drug: Artemether Lumefantrine (ALU) and Injection Artesunate. For prevention, participants earned one point for mentioning bed nets and half a point for other measures such as the use of mosquito coils, wearing long clothes, insecticide spray, weeding, or sewage disposal. Knowledge levels were classified as high, average, or low. Participants scoring above 50% were considered knowledgeable (good knowledge), while those scoring below 50% were regarded as not knowledgeable (poor knowledge).^17, 18^

### Ethical Considerations

Confidentiality was maintained throughout the study, and participation of both schoolchildren and parents and caretakers was voluntary. All schoolchildren gave their assent, while all caretakers provided informed consent. Participants had the freedom to withdraw from the study at any time, even after signing either the assent or consent forms. Ethical clearance for the study was granted by the Muhimbili University of Health and Allied Sciences (MUHAS) with the research clearance certificate number MUHAS-REC-12-2022-1490.

## RESULTS

### Characteristics of Study Participants

**FIGURE 1:**
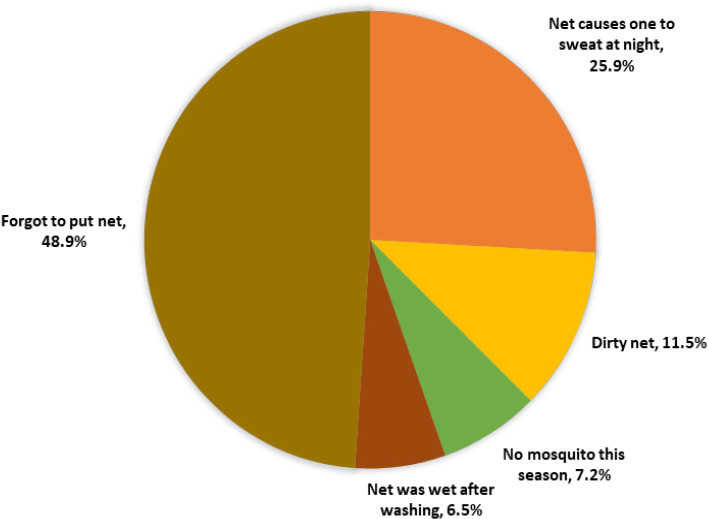
Reported Reasons for Not Sleeping Under a Bed Net (N = 139)

A total of 530 schoolchildren participated in the study, with Masuguru and Kwabutu primary schools (urban) contributing 47.7% and 15.7% of participants, and Heinkele and Matombo primary schools (rural) comprising 19% and 17.6%, respectively. The majority of children (96%) were under 14 years of age, with a median of 12 years (IQR: 11–13), and most were female (53.4%) (Table 1). Of all schoolchildren, 462 (87.2%) reported owning a mosquito net at home, and among these, 323 (69.8%) had used it the night before the interview. The main reasons reported by those who did not use nets included the net causing sweating at night, dirty nets, lack of mosquitoes during the season, or the net being wet after washing (Figure 1). Household visits were conducted for 110 children, predominantly from Masuguru village (48.2%), and over 90% of houses had iron sheet roofs, and 30% had mud walls. Household heads were mainly farmers (53%) and had primary education (56.4%) (Table 2).

**TABLE 1:** Demographic Characteristics of Schoolchildren Involved in the Study (N = 530)

Characteristic	Frequency (N)	Percentage (%)
Age range (years)		
10 – 14	510	96.2
15 – 18	20	3.8
Gender		
Male	247	46.6
Female	283	53.4
Selected Primary schools		
Masuguru	253	47.7
Heinkele	101	19.0
Matombo	93	17.6
Kwabutu	83	15.7
School locality		
Town	336	63.4
Rural	194	36.6

**TABLE 2:** Characteristics of Households’ Head (N = 110)

Characteristic	Frequency (N)	Percentage (%)
Village name		
Masuguru	53	48.2
Heinkele	20	18.2
Kwabutu	17	15.5
Matombo	20	18.1
Education level of head of household		
No education	6	5.4
Primary	62	56.4
Secondary & above	42	38.2
Occupation of head of household		
Formal employment	12	10.9
Entrepreneur	17	15.4
Farmer	58	52.7
Other	23	21.0
House roofing materials		
Iron sheet	103	93.6
Grass	7	6.4
Nature of house walls		
Block	77	70
Mud	33	30
Average monthly Income (Tsh)		
≤ 50,000	42	38.2
> 50,000	68	61.8
Number of individuals in household		
2 – 4	28	25.5
5 – 6	62	56.3
Above 7	20	18.2
Source of Mosquito Net		
Government Aid	91	82.7
Bought from shop	19	17.3

### Level of Malaria knowledge among schoolchildren

All 530 schoolchildren reported having heard of malaria as a disease (Table 3). Overall, 480 schoolchildren (90.6%) had good knowledge about malaria in terms of modes of transmission, symptoms, prevention, and treatment methods. The results for each category were as follows.

- **Knowledge of the mode of malaria transmission:** About 437 (82.45%) children correctly identified mosquito bites as the mode of malaria transmission. Other transmission methods were reported by 59 (11.13%) children, which included drinking dirty water (3.02%), eating contaminated food (2.83%), coughing (1.89%), touching vomitus or blood of an infected person (1.51%), sharing plates with an infected person (0.75%), sharing sharp objects (0.57%), and human bites (0.57%). Only 34 (6.42%) did not know how malaria is transmitted (Table 3).
- **Knowledge of the symptoms of malaria:** The majority of schoolchildren in Muheza district had good knowledge of malaria symptoms. About 405 (76.42%) children were able to report fever and headache as common symptoms of malaria, whereas 39 (7.36%) reported diarrhoea and vomiting as disease symptoms. About 43 (8.11%) reported other symptoms, which included nausea, loss of appetite, body weakness, dizziness, feeling cold, and abdominal pain (Table 3).
- **Knowledge of Malaria treatment methods:** Children were asked if they were aware of drugs used for malaria treatment. The majority (485, 91.5%) correctly reported Artemether Lumefantrine (ALU) as the drug for malaria treatment. About 16 (3.0%) children reported herbal drugs as the preferred treatment option. The rest of the children, 29 (5.47%), were unaware of drugs used for malaria treatment (Table 3).
- **Knowledge of Malaria prevention methods:** Using mosquito nets was the most commonly reported method of malaria prevention, reported by 464 children (87.55%). Children who were unaware of any of the prevention strategies were about 37% (7). About 29 (5.47%) children reported other preventive methods, which included cleaning surrounding environments, burning or spraying mosquito repellents, and wearing long-sleeved clothes (Table 3).

**TABLE 3:** Schoolchildren's Knowledge of Malaria Transmission, Symptoms, Treatment, and Prevention Methods (N = 530)

Variable	Category	Frequency (N)	Percentage (%)
Have you ever heard about malaria?	Yes	530	100
How is malaria transmitted?	Mosquito bite	437	82.45
	Other	59	11.13
	Don't know	34	6.42
What symptoms does a person with malaria have?	Headache and fever	405	76.42
	Diarrhea and vomiting	39	7.36
	Other symptoms	43	8.11
	Don't know	43	8.11
What methods can one use to protect against malaria?	Don't know	37	6.98
	Other methods	29	5.47
	Using mosquito nets	464	87.55
What drugs do you know are used for treatment of Malaria?	Artemether Lumefantrine (ALU)	485	91.51
	Herbal medicine	16	3.02
	Don't know	29	5.47

### Factors associated with mosquito net use among schoolchildren

The bivariate analysis showed that children who lived in urban residences were 1.67 times more likely to use mosquito nets compared to children living in rural areas (PR 1.67; 95% CI, 1.44 to 1.95). Similarly, children who owned a TV or radio at home were 2 times more likely to use mosquito nets compared to children who had no TV/radio (PR 2.00; 95% CI, 1.47 to 2.70). Similarly, children who lived with their parents were 1.7 times more likely to use mosquito nets than those living with other care providers (PR 1.70; 95% CI, 1.42 to 1.97) (Table 4).

**TABLE 4:** Bivariate and Multivariable Analysis of Determinants of Mosquito Net Use Among Schoolchildren in Muheza District (N = 530)

Characteristic	Used Net		Bivariate analysis		Multivariable analysis	
	N	n (%)	^*^PR (95% Cl)	*p-value*	^*^aPR (95% Cl)	*p-value*
Age (years)						
10 – 14	510	357 (70)	1.08 (0.62 – 1.87)	.79		
15 – 19	20	13 (65)	1			
Residence						
Town	336	275 (82)	1.67 (1.44 – 1.95)	<.001	1.39 (1.19 – 1.62)	<001
Rural	194	95 (49)	1			
Class level						
Standard V	160	108 (68)	1			
Standard VI	177	111 (63)	0.93 (0.79 – 1.09)	.36		
Standard VII	193	151 (78)	1.16 (1.02 – 1.32)	.24		
Sex						
Male	248	168 (68)	1			
Female	282	202 (72)	1.06 (0.94 – 1.18)	.33		
Parent living with						
Biological	359	288 (80)	1.70 (1.42 – 1.97)	<.001	1.35 (1.14 – 1.58)	<001
Other caregiver	171	82 (48)	1			
TV/radio ownership						
Yes	458	343 (75)	2.00 (1.47 – 2.70)	<.001	1.39 (1.05 – 1.83)	.02
No	72	27 (38)	1			

* PR = Crude Prevalence ratio, aPR = Adjusted Prevalence Ratio, CI = Confidence Interval, N = Total in category, n = Number who used a net

On the other hand, we analysed the household factors to determine their association with mosquito net use among schoolchildren in Muheza district, as shown in Table 5. In bivariate analysis, we found that children whose parents had an average monthly income of above 50,000/= Tsh were 1.6 times more likely to use mosquito nets compared to children whose parents earned a monthly income of less than or equal to 50,000/= Tsh (PR 1.59; 95% CI, 0.94 to 2.71). Also, children who lived in block houses were two times more likely to sleep under mosquito nets compared to children living in muddy houses (PR 2.22; 95% CI, 1.34 to 3.67). On the other hand, children living in houses with electricity were about 4 times more likely to sleep under mosquito nets compared to those whose houses had no electricity (PR 3.71; 95% CI, 1.67 to 8.23). Nevertheless, children in households where the ratio of family members to bed nets was ≤ 2 were approximately 4 times more likely to use mosquito nets compared to those in households with a ratio > 2 (PR 3.60; 95% CI, 2.00 to 6.50). Likewise, children with mosquito nets aged ≤ 3 years were 2.78 times more likely to sleep under them compared to those with nets older than 3 years (PR 2.78; 95% CI, 1.90 to 4.10).

**TABLE 5:** Bivariate and Multivariate Analysis of Household Factors Influencing Mosquito Net Use Among Schoolchildren in Muheza District (N = 110)

Characteristic	Used Net		Bivariate analysis		Multivariable analysis	
	N	n (%)	*PR (95% Cl)	*p-value*	*aPR (95% Cl)	*p-value*
Education level household head						
No education	6	1 (17)	1			
Primary education	62	41 (66)	4.0 (0.65 – 24.15)	.14	2.72 (0.76 – 9.80)	.12
Secondary & above	42	26 (62)	3.71 (0.50 – 27.37)	.20	2.61 (0.72 – 9.48)	.14
Occupation head household						
Formal employment	12	6 (50)	1			
Entrepreneur	17	U (65)	1.29 (0.66 – 2.53)	.45		
Farmer	58	34 (59)	1.17 (0.64 – 2.14)	.61		
Other	23	17 (74)	1.48 (0.80 – 2.74)	.22		
Household head monthly income (Tsh)						
≤50,000	42	19 (45)	1			
>50,000	68	49 (72)	1.59 (0.94 – 2.71)	.01	1.19 (0.91 – 1.55)	20
House roofing materials						
Iron sheet	103	64 (62)	1.09 (0.56 – 2.11)	.80		
Grass	7	4 (57)	1			
House wall materials						
Block	77	57 (74)	2.22 (1.34 – 3.67)	<.01	1.36 (0.93 – 1.98)	.12
Mud	33	U (33)	1			
House has Electricity						
Yes	85	63 (74)	3.71 (1.67 – 8.23)	<01	2.17 (1.12 – 4.20)	.02
No	25	5 (20)	1			
Family size to bed net ratio						
≤2	71	59 (83)	3.60 (2.00 – 6.50)	<.01	2.30 (1.36 – 3.88)	<.01
>2	39	9 (23)	1			
Age of mosquito net since first use						
≤3 years	55	50 (91)	2.78 (1.90 – 4.10)	<.01	1.69 (1.19 – 2.41)	<.01
>3 years	55	18 (33)	1			

In multivariate modified Poisson regression analysis, we found that residing in an urban residence (aPR 1.40; 95% CI, 1.19 to 1.62), living with a biological parent (aPR 1.35; 95% CI, 1.14 to 1.58) and owning a TV or radio (aPR 1.39, CI, 1.05 to 1.83) remained statistically significant. Conversely, a multivariate modified Poisson regression analysis showed that houses with electricity (aPR 2.17; 95% CI, 1.12 to 4.20), families with a household size to bed net ratio ≤ 2 (aPR 2.30; 95% CI, 1.36 to 3.88) and having a mosquito net ≤ 3 years old (aPR 1.69; 95% CI, 1.19 to 2.41) remained statistically significant (Table 4).

## DISCUSSION

This study assessed the level of malaria knowledge and the factors associated with mosquito net use among schoolchildren in Muheza district. We found a high level of malaria knowledge in this population. Factors significantly associated with mosquito net use included living in urban areas, residing with parents, having a TV at home, family size to mosquito net ratio of less than or equal to two, living in homes with electricity, and using mosquito nets that were three years old or less since first use. Each finding was discussed as follows.

### Level of malaria knowledge among schoolchildren in Muheza District

Our research revealed that every schoolchild recognized malaria as an illness, with the majority linking it to mosquito bites. Some had misunderstandings about how malaria spreads, such as linking it to consuming unclean food or water, sharing sharp objects, touching infected blood or vomit, and sleeping near an infected person. Similar misconceptions were reported by other studies conducted in Tanzania^19, 20^, Uganda ^21^, Burundi ^22^, Nigeria ^23^, Cameroon^24^, Côte d’Ivoire^25^, Ghana^14^, and India.^26^ Shared cultural beliefs and practices across these regions may have contributed to the observed similarities in disease misconceptions.

Conversely, mosquito net use is a well-known method of prevention of malaria mentioned by the majority of schoolchildren in Muheza district. Similar results were reported by studies conducted in Tanzania ^19^, Ethiopia ^27^, Cameroon ^24^, and Senegal.^18^ Since malaria is endemic in the majority of these countries with mosquito nets being the major disease prevention method advocated by the WHO in disease-endemic areas, therefore this can explain the similarity in the findings.

Artemether Lumefantrine (ALU) was the main antimalarial drug reported by most schoolchildren, however, some children still believed in herbal medicine for treatment. Similar beliefs have been reported in a study that was conducted in school adolescents in Nigeria where it was reported that the adolescents would consult herbalists or use local herbs ^23^, when they get infected with malaria. Herbal medicine has also been reported to be used by communities through studies conducted in Tanzania ^20^, Uganda ^21^, and India.^26^ As these beliefs are transmitted across generations, more efforts are needed to break this misconception in the communities. On the other hand, a high level of awareness of ALU may have been attributed to a previous study conducted 3 years ago that involved the provision of the drug to schoolchildren in the area.

### Factors associated with mosquito net use among schoolchildren

Our study found that schoolchildren living with their parents were more likely to use mosquito nets than those living with other caregivers. This finding aligns with studies conducted in Nigeria ^28^ and Burkina Faso ^29^, which similarly report higher mosquito net usage among children residing with their parents. Additionally, a study from Ethiopia highlights that biological parents are more likely to provide support and care ^30^, and therefore ensuring access to preventive measures such as mosquito nets. These consistent findings across different contexts emphasize the importance of parental care in malaria prevention among children.

Similarly, our study observed a significant association between mosquito net use and residing in urban settings. This finding is consistent with a nationwide school malaria parasitemia survey conducted in Tanzania, which reported that schoolchildren living in urban areas had higher odds of using mosquito nets compared to those in rural areas.^10^ Similar findings were reported in different studies from Rwanda^31^, Ethiopia^4,32^, Somalia^33^ and Cameroon.^34^ As parents/caregivers and children in urban settings are more likely to have easy access to mosquito nets^31^, and good exposure to different malaria health information, this may explain the observed similarities in the findings. On the other hand, having similar rural settings in the majority of sub-Saharan countries, most of which have poor infrastructure to allow access to health services, may contribute to the observed similarities in the findings. Not only that, as it has been reported that staying in areas with health facilities increases chances of using mosquito nets among schoolchildren.^35^ Thus, the greater presence of health facilities in urban areas likely contributes to higher mosquito net use in these.

The study also found that possessing a mosquito net used for less than three years was significantly associated with increased likelihood of its use among schoolchildren. Our finding is in line with a study conducted in Ethiopia ^32^, where it was found that increasing mosquito net age was associated with a lower likelihood of its use. The reduced use of older nets may be due to increased damage and decreased quality over time, leading to decreased effectiveness. This may be related to the fact that as the age of the mosquito net increases, it becomes more susceptible to wear and tear, which can reduce its effectiveness over time, highlighting the importance of regular maintenance and timely replacement of mosquito nets to ensure they continue to provide effective protection for vulnerable populations, including school children.

Conversely, this study suggests a crucial role of electricity access in promoting mosquito net use among schoolchildren. This finding is supported by studies that were conducted in Rwanda ^36^ and Mozambique ^37^, which reported that access to electricity was associated with increased likelihood of mosquito net use. It has also been noted that communities without electricity struggle to access timely health-related information, as they often face difficulties in powering devices like radios due to the frequent purchase of batteries ^38^, a barrier that limits their ability to stay informed and adopt preventive measures. Thus, household electrification not only improves living conditions but also enhances malaria prevention efforts by facilitating greater information dissemination and awareness among children.

Our study findings also suggest an association between mosquito net use and living in houses whose household size to bed net ratio is ≤2. Similar findings have also been reported in different studies conducted in Ethiopia ^4,30^, and the Democratic Republic of Congo.^39^ The observed similarities among the studies are in line with the WHO recommendations, which further reinforce that maintaining a bed net ratio of one net for every two persons is essential to maximize community-wide benefits and reduce transmission risk.^40^

This study found no significant association between the education level of the head of household and mosquito net use among schoolchildren in the study area. Similar findings have been reported in Rwanda ^36^ and Mozambique.^41^ However, contrasting results were observed in a nationwide school malaria parasitemia survey in Tanzania ^10^, and a study in Nigeria ^28^, where higher parental education was linked to increased net use. These inconsistencies may reflect differences in sample size, study context, or time periods. While the association in our study was not statistically significant, reporting such findings is important to build a comprehensive understanding of predictors of mosquito net use.

## CONCLUSION

Although there was evidence of adequate malaria knowledge among schoolchildren in Muheza district, 30% didn't use mosquito nets the previous night, and 3% believed in herbal remedies for malaria treatment. Urban residence, parental care, TV or radio ownership, access to electricity, a family-to-bed-net ratio of ≤ 2, and owning a net of under 3 years since its initial use were linked to net usage. It is vital to strengthen malaria intervention and education programs that focus on increasing awareness, improving access to preventive methods, and correcting misconceptions, especially in rural areas where such misconceptions persist.
